# Indicators of transparency and data sharing in scientific writing in published randomized controlled trials in orthodontic journals between 2019 and 2023: an empirical study.

**DOI:** 10.1093/ejo/cjae064

**Published:** 2024-11-21

**Authors:** Sophie Schueller, Filippos Mikelis, Theodore Eliades, Despina Koletsi

**Affiliations:** Clinic of Orthodontics and Pediatric Dentistry, Center of Dental Medicine, University of Zurich, Zurich, Switzerland; Department of Orthodontics, School of Dentistry, National and Kapodistrian University of Athens, Athens, Greece; Clinic of Orthodontics and Pediatric Dentistry, Center of Dental Medicine, University of Zurich, Zurich, Switzerland; Clinic of Orthodontics and Pediatric Dentistry, Center of Dental Medicine, University of Zurich, Zurich, Switzerland; Meta-Research Innovation Center at Stanford (METRICS), Stanford University, California, CA, United States

**Keywords:** data sharing, individual participant data, registration, transparency, orthodontic RCTs

## Abstract

**Aim:**

To identify data sharing practices of authors of randomized-controlled trials (RCTs) in indexed orthodontic journals and explore associations between published reports and several publication characteristics.

**Materials and methods:**

RCTs from indexed orthodontic journals in major databases, namely PubMed® (Medline), Scopus®, EMBASE®, and Web of Science™, were included from January 2019 to December 2023. Data extraction was conducted for outcome and predictor variables such as data and statistical code sharing practices reported, protocol registration, funding sources, and other publication characteristics, including the year of publication, journal ranking, the origin of authorship, number of authors, design of the RCT, and outcome-related variables (e.g. efficacy/safety). Statistical analyses included descriptive statistics, cross-tabulations, and univariable and multivariable logistic regression.

**Results:**

A total of 318 RCTs were included. Statement for intention of the authors to provide their data upon request was recorded in 51 of 318 RCTs (16.0%), while 6 of 318 (1.9%) openly provided their data in repositories. No RCT provided any code or script for statistical analysis. A significant association was found between data sharing practices and the year of publication, with increasing odds for data sharing by 1.56 times across the years (odds ratio [OR]: 1.56; 95% confidence interval [CI]: 1.22, 2.01; *P* < .001). RCTs reporting on safety outcomes presented 62% lower odds for including positive data sharing statements compared to efficacy outcomes (OR: 0.38; 95% CI: 0.17, 0.88). There was evidence that funded RCTs were more likely to report on data sharing compared to non-funded (*P* = .02).

**Conclusions:**

Albeit progress has been made towards credibility and transparency in the presentation of findings from RCTs in orthodontics, less than 20% of published orthodontic trials include a positive data sharing statement while less than 2% openly provide their data with publication.

## Introduction

There is an increasing interest of researchers in transparent research practices that ensure credible and reproducible research [1, 2]. The observable trend is towards open science practices, encompassing various practices such as study registration, protocol accessibility, data sharing of anonymized individual participant data (IPD), and code sharing. Evidence substantiates the benefits of open science for research endeavours, researchers themselves, and the broader research community [3].

Accessibility to the data, analytical code, and supporting materials enables researchers to refine methods, validate findings, explore unanticipated or emerging research questions, and expedite research by integrating existing datasets [4]. However, early empirical findings suggest that a vast number of scientific publications lack reproducibility or are inflated [5]. This problem extends to research in orthodontics, following current practices across biomedicine [6].

Therefore, it is key for credible research that anonymous IPD, statistical methods, and analytical code or statistical scripts are made publicly available [7]. In some biomedicine disciplines, for example, genomics, data sharing is already the norm [8]. However, in most medical and health publications, transparency, and reproducibility indicators like data or code sharing, are at a low level, with numbers for code sharing reported to be scarce [9, 10]. Furthermore, a discrepancy between data-sharing declarations in publications and actual data availability has been documented [9].

To promote open science practices, the FAIR (Finding, Accessible, Interoperable, Reusable) principles have been formulated. These aim to support researchers in making data more reusable and thus further increase the validation of research attempts and reproducibility [11]. Furthermore, some biomedical journals have already made data sharing mandatory for their publication of clinical trials (e.g. *PLoS* and *the British Medical Journal*) [12]. More initiatives are likely to follow, since for example, the US government proposed to its federal funding agencies to implement policies that require state-funded research to be made publicly available by the end of 2025 [13].

Data requirements from clinical trials are central to evidence-based medicine; however, data sharing is rather uncommon [11]. As evidence-based orthodontics has evolved, researchers have prioritized study design and quality, leading to a significant increase in performing and publication of RCTs in orthodontics over the past decade [14]. In line with other biomedical disciplines, RCTs in orthodontics, lack credibility, reproducibility, and transparency, and therefore this increases the risk for wasteful research practices. Estimates suggest that reporting issues in orthodontic clinical research have been leading to over 80% of avoidable waste [15].

To improve the credibility of reporting, pre-registration can increase the transparency of studies. The International Committee of Medical Journal Editors encourages editors to publish only pre-registered clinical trials [16]. Prior research showed significant reporting biases, where studies that were not registered reported more statistically significant findings than studies that were registered a priori [17–21]. Randomized trials in orthodontics have been reported to lack credibility due to methodological shortcomings [6, 17, 20, 21]. In terms of transparency, the first empirical studies in dentistry have revealed that RCT registrations range between 34% and 56%, however, demonstrated low values for data and code sharing [22]. Compared to periodontics, orthodontic RCTs have been reported to have significantly lower levels of trial registration (34% vs. 56%) and open data provision (2.7% vs. 7.4%) [19]. Furthermore, the sharing of open data in dental RCTs is not yet widely recognized as an essential part of the scientific process. However, findings indicate that most patients participating in RCTs are in favour of sharing the data of the trials [23, 24] and evidence suggests that data sharing is also linked to higher citation rates [25].

In all, the scientific contribution and clinical importance of this work largely lie in the documented significance of the disseminated information for decision-making in clinical practice. This relates to how the benefits and risks of certain interventions are considered and the level of trust one may place in pooled evidence stemming from meta-analyses incorporating results of RCTs. If the primary studies’ evidence cannot be replicated, transparency is severely harmed, and validity cannot be confirmed. To this extent, this is bound to be highly influential for the society and public health.

This study aimed to identify transparency indicators as well as to record the reported data sharing practices by authors of RCTs in orthodontic publications during the last 5 years, from 2019 to 2023. Moreover, we intended to examine if there were any associations between published reports and publication characteristics such as geographic location, number of authors, study design, outcome-related variables, sources of funding, registration practices, and data sharing. The null hypothesis is that these variables have no significant effect on data sharing practices.

## Materials and methods

### Protocol registration

The protocol of this empirical study was registered at the Open Science Framework and the underlying data are openly available in an anonymized manner [https://osf.io/avt5b/].

### Eligibility criteria and data extraction

The sample consisted of RCTs published in English language in major orthodontic journals, over the last 5 years, namely between 2019 and 2023. The target journals were indexed in major databases, namely PubMed® (Medline), Scopus®, EMBASE®, and Web of Science™, while cite scores or impact factors of the journals were not considered as inclusion criteria. Eligible journals were: the *American Journal of Orthodontics and Dentofacial Orthopedics (AJODO), Angle Orthodontist (ANGLE), APOS Trends in Orthodontics (APOS), Australasian Orthodontic Journal (AOJ), Clinical and Investigative Orthodontics (formerly known as Orthodontic Waves) (CIO), Dental Press Journal of Orthodontics (DENTAL PRESS), European Journal of Orthodontics (EJO), International Orthodontics (IO), Journal of Orofacial Orthopedics (JOOF), Journal of Orthodontics (JO), Journal of the World Federation of Orthodontists (JWFO), Korean Journal of Orthodontics (KJO), Orthodontics and Craniofacial Research (OCR), Progress in Orthodontics (PROGRESS), Seminars in Orthodontics (SEMINARS),* and *Turkish Journal of Orthodontics (TJO).*

All articles published in these 16 journals were screened electronically to identify RCTs published between 1 January 2019, and 31 December 2023. Titles and abstracts of all articles within this period were screened by two researchers and all studies identified as RCTs involving humans were included. When in doubt about eligibility, the full text was retrieved, and the entire article was screened. Pilot studies as well as RCTs involving animals were excluded.

Data extraction was performed independently by two investigators (SS, FM) following initial piloting for all eligible RCT full-text articles using customized, standardized forms. A third investigator resolved any disagreement until a consensus was reached (DK). Data extraction included the following variables:

- Name of the journal
- Publication year
- Quantile Journal Ranking
- Continent of corresponding author (America, Europe, Asia/other)
- Number of authors
- Number of centre(s) (single-centre or multi-centre)
- Study design (split-mouth, parallel, cross-over, or factorial)
- Number of groups
- Sample size
- Type of primary outcome (efficacy or safety)
- Statistical significance of the primary outcomes
- Sources of funding (state/university, industry, state/university, and industry funding)
- Trial registration (protocol)
- Data sharing intentions in registration (status provided in the protocol)
- Data sharing and statistical scripts or code sharing statements

The primary outcome comprised the proportion of randomized controlled trials (RCTs) including positive data availability statements compared to papers not including any intention or reporting of data sharing. Data sharing practices documentation comprised data sharing statements of ‘sharing upon request’ within the paper, published anonymized data of individual participants that had already been made available in an open repository, or any other means by which the raw data were made accessible.

### Statistical analysis

Descriptive statistics for the examined variables and cross-tabulations for the likelihood of RCTs reporting of data sharing across the examined variables were performed. Univariable and multivariable logistic regression models were applied to examine the association between year, quantile journal ranking, continent of authorship, number of authors, design of the RCT, number of centre(s), type and significance of outcome, funding, trial registration, and RCT-reported data sharing. All predictors were initially included in the multivariable model and backward elimination of non-significant predictors (deletion criterion *P* > .10) was employed. The Hosmer–Lemeshow test was used to check model fit. Results were presented as absolute numbers and percentages and as effect sizes with a 95% confidence interval (95% CI), and *P* values, where applicable. The unweighted kappa statistic was used to assess inter-rater agreement on the outcomes of interest. A kappa value of 0.87 (95% CI: 0.62, –1.00) was achieved indicating perfect agreement. The predefined level of significance was set at *P* < .05 (two sided). All analyses were conducted with Stata (version 15.1; Stata Corporation, College Station, TX, USA).

## Results

**Figure 1.**
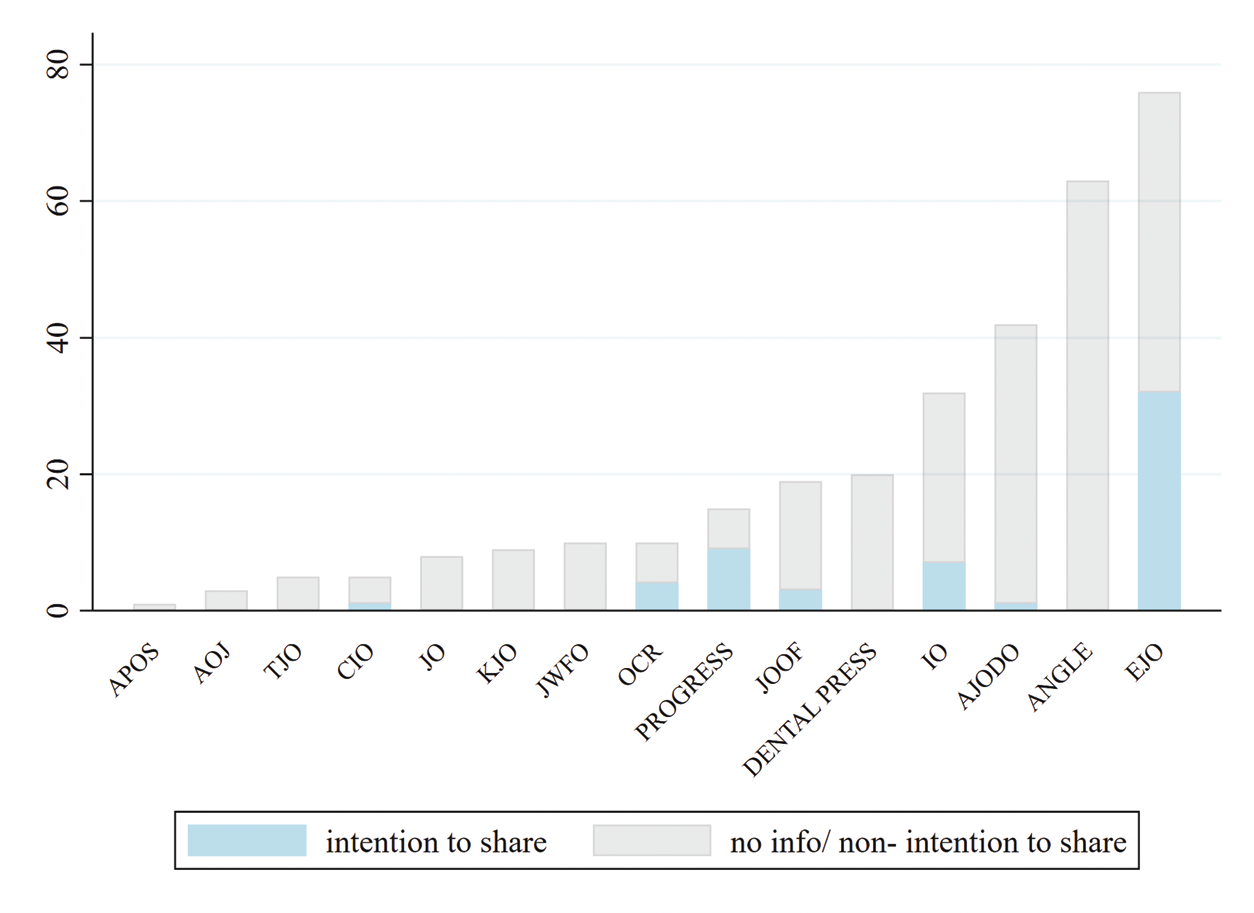
Bar graph of data sharing practices of RCTs across all indexed orthodontic journals.

Our sample included 318 RCTs, with 57 (17.9%) of those either including a positive data sharing statement reflecting the intention of the authors to provide their data upon request (51/318; 16.0%) or openly providing their data in repositories in the form of anonymized IPD (6/318; 1.9%). No published report provided any indication of sharing the code or script used to analyse their data. One of the eligible journals, that is *SEMINARS*, was not represented since, following its scope, no RCTs were published. The distribution of RCTs across the years assessed was relatively even, while there was a higher percentage of RCTs documented with positive intention to share data, across the last 3 years (2021–2023), with a percentage range of 20.0%–26.1% compared to earlier publications of RCTs that appeared mostly negative to adopting such practices (~6.5%). The journals that contributed mostly to this sample were the *EJO* with 76 of 318 RCTs (23.9%), the *ANGLE* (63/318; 19.8%), and the *AJODO* (42/318; 13.2%). The *EJO* presented the highest number of studies with positive data sharing statements (32/76; 42.1%), followed by *PROGRESS* which contributed a total of 15 RCTs, 9 of which included positive data sharing statements (60.0%) (Fig. 1). Most RCTs were published in journals that belonged to the Q1 (highest) ranking (67.6%), mostly by non-European and non-American authors (186/318; 58.5%), not including a methodologist (282/318; 88.7%), being conducted at a single centre (276/318; 86.8%) and of parallel design (242/318; 76.1%). Four to five authors were more frequently involved in authorship (134/318; 42.1%). Outcomes assessed were mostly efficacy in nature (233/318; 73.3) and statistically significant (188/318; 59.1%). Most RCTs were not funded or did not include related information (167/318; 52.5%), while 106 of 318 (33.3%) were university-funded or sponsored by state institutions. Studies funded by any source more frequently reported a positive data-sharing statement (26.5%). The majority were registered (195/318; 61.3%), while doubling the percentage of registered RCTs included positive data-sharing statements compared to non-registered (43/195; 22.0% vs. 14/123; 11.4%) (Table 1). A list of all registries reported across the present sample of RCTs is provided in Supplementary Table 1. In addition, editorial policies regarding requirements for data availability statements for all included journals are presented in Supplementary Table 2.

**Table 1.** Characteristics of RCTs, by data sharing intention statement (318).

	Intention to share data (positive statement)		
	No *N* (%)	Yes *N* (%)	Total *N* (100 %)
Year			
2019	54 (93.1)	4 (6.9)	58
2020	44 (93.6)	3 (6.4)	47
2021	60 (80.0)	15 (20.0)	75
2022	48 (73.9)	17 (26.1)	65
2023	55 (75.3)	18 (24.7)	73
Journal			
* American Journal of Orthodontics and Dentofacial Orthopedics*	41 (97.6)	1 (2.4)	42
* Angle Orthodontist*	63 (100.0)	0 (0.0)	63
* APOS Trends in Orthodontics*	1 (100.0)	0 (0.0)	1
* Australasian Orthodontic Journal*	3 (100.0)	0 (0.0)	3
* Clinical and Investigative Orthodontics (formerly known as Orthodontic Waves)*	4 (80.0)	1 (20.0)	5
* *	20 (100.0)	0 (0.0)	20
* European Journal of Orthodontics*	44 (57.9)	32 (42.1)	76
* International Orthodontics*	25 (78.1)	7 (21.9)	32
* Journal of Orofacial Orthopedics*	16 (84.2)	3 (15.8)	19
* Journal of Orthodontics*	8 (100.0)	0 (0.0)	8
* Journal of the World Federation of Orthodontists*	10 (100.0)	0 (0.0)	10
* Korean Journal of Orthodontics*	9 (100.0)	0 (0.0)	9
* Orthodontics and Craniofacial Research*	6 (60.0)	4 (40.0)	10
* Progress in Orthodontics*	6 (40.0)	9 (60.0)	15
* Seminars in Orthodontics*	0 (0.0)	0 (0.0)	0
* Turkish Journal of Orthodontics*	5 (100.0)	0 (0.0)	5
Journal quantile ranking			
Q1 (Top 25%)	173 (80.5)	42 (19.5)	215
Q2–Q4	88 (85.4)	15 (14.6)	103
Continent			
America	34 (94.4)	2 (5.6)	36
Europe	70 (72.9)	26 (27.1)	96
Asia/Other	157 (84.4)	29 (15.6)	186
No. of authors			
1–3	73 (84.9)	13 (15.1)	86
4–5	107 (79.6)	27 (20.2)	134
≥6	81 (82.7)	17 (17.3)	98
Methodologist			
No	230 (81.6)	52 (18.4)	282
Yes	31 (86.1)	5 (13.9)	36
Design			
Parallel	199 (82.2)	43 (17.8)	242
Split mouth	54 (84.4)	10 (15.6)	64
Cross over	8 (66.7)	4 (33.3)	12
Centre			
Single	227 (82.3)	49 (17.7)	276
Multi	34 (81.0)	8 (19.0)	42
Type of outcome			
Efficacy	184 (79.0)	49 (21.0)	233
Safety	77 (90.6)	8 (9.4)	85
Statistical significance of outcome			
No	105 (80.8)	25 (19.2)	130
Yes	156 (83.0)	32 (17.0)	188
Funding			
None/No information	150 (89.8)	17 (10.2)	167
Industry/Company	20 (64.5)	11 (35.5)	31
University/State	82 (77.4)	24 (22.6)	106
Both	9 (64.3)	5 (35.7)	14
Registration			
No	109 (88.6)	14 (11.4)	123
Yes	152 (78.0)	43 (22.0)	195
Total	261 (82.1)	57 (17.9)	318

A cross-tabulation between the intention to share practices documented in the registered protocol and data sharing statements in the final published RCT report did not reveal any clear association (Fisher’s exact test, *P* = .21). Only in a third of cases were intention to share statements in the protocol (33.3%) followed by definite data sharing plans in the published report (Table 2). Furthermore, a linear trend for the effect of the year on the adoption of data sharing statement plans was confirmed (test for trend, *P* = .001) (Table 2).

**Table 2.** Actual reporting of data sharing in the publication report against the intention to share statement at the registered protocol (*n* = 195).

	Data sharing statement in the publication report (positive statement)			*P* value^a^
	No *N* (%)	Yes *N* (%)	Total *N* (100 %)	
Intention to share data (in registered protocol)				.21
No reporting- no information	70 (84.3)	13 (15.7)	83	
Intention to share statement (+)	12 (66.7)	6 (33.3)	18	
No intention to share (-)	52 (73.2)	19 (26.8)	71	
Plan to disclose at a later stage/undecided	18 (78.3)	5 (21.7)	23	
Total	152 (77.9)	43 (22.1)	195 (100.0)	

^a^Fisher’s exact test.

According to the multivariable regression model, for each more recent publication year since 2019, there was strong evidence of a significant effect on positive data sharing practices (odds ratio, OR: 1.56; 95% CI: 1.22, 2.01; *P* < .001). RCTs reporting on safety outcomes presented 62% lower odds for including positive data sharing statements compared to efficacy outcomes (OR: 0.38; 95% CI: 0.17, 0.88). Finally, there was evidence that funded RCTs were more likely to report on data sharing compared to non-funded (Wald global test, *P* = .02) (Table 3).

**Table 3.** Univariable and multivariable logistic regression with odds ratios and respective confidence intervals for the effect of year, journal quantile ranking, continent (geographical affiliation), no. of authors, design, no. of centres, type of outcome, funding, and registration on data sharing statement (*n* = 318).

Category	Univariable			Multivariable		
	OR	95% CI	*P* ^a^	OR	95% CI	*P* value^a^
Year			0.001			<.001
Per unit	1.47	1.18, 1.85		1.56	1.22, 2.01	
Journal quantile ranking			0.28			
Q1 (top 25%)	Reference					
Q2–Q4	0.70	0.37, 1.34				
Continent			0.01^a^			.10^a^
America	Reference			Reference		
Europe	6.31	1.42, 28.17		4.99	1.03, 24.02	
Asia/Other	3.14	0.71, 13.80		3.09	0.68, 14.13	
No. of authors			0.63^a^			
1–3	Reference					
4–5	1.42	0.69, 2.93				
≥6	1.18	0.54, 2.59				
Design			0.36^a^			
Parallel	Reference					
Split mouth	0.86	0.40, 1.82				
Cross over	2.31	0.67, 8.03				
Methodologist			0.50			
No	Reference					
Yes	0.71	0.26, 1.92				
Center			0.84			
Single	Reference					
Multi	1.09	0.48, 2.50				
Type of outcome			0.02			.02
Efficacy	Reference			Reference		
Safety	0.39	0.18, 0.86		0.38	0.17, 0.88	
Statistical significance			0.61			
No	Reference					
Yes	0.86	0.48, 1.54				
Funding			<0.001^a^			.02^a^
None/No information	Reference			Reference		
Industry/Company	4.85	1.99, 11.82		2.76	0.98, 7.77	
University/State	2.58	1.31, 5.08		2.82	1.39, 5.74	
Both	4.90	1.47, 16.32		3.13	0.82, 12.00	
Registration			0.02			
No	Reference					
Yes	2.20	1.15, 4.22				

^a^Wald test for overall statistical significance.

## Discussion

Data and code sharing in academic research refer to the practice that authors share their anonymized IPD, as well as the code for statistical analysis openly and publicly [26]. When data are openly available, it allows other researchers to verify or re-analyse the data, which enhances the consistency and ultimately ensures the validity of the study’s findings.

This study aimed to record and assess data sharing practices across all currently indexed orthodontic journals, spanning over the most recent five years and including RCTs until the end of 2023. Moreover, in this study, we examined whether potential predictor variables and publication characteristics have an impact on data sharing practices. Given the identification of significant associations between publication characteristics and data sharing practices, the null hypothesis was rejected.

Our findings reveal that despite the increasing emphasis on transparency in scientific research, the adoption of data sharing practices in orthodontic RCTs remains low. However, our study highlights that in orthodontic research, data sharing practices may be more common than in other oral health disciplines [22, 27]. Orthodontics is not only at the forefront of oral health science in terms of data sharing but may also be considered one of the leading disciplines in addressing other methodological issues that have been identified as affecting the quality and rigour of research in general, even though the necessity for improvement remains [28]. The variability in data sharing practices identified across orthodontic journals, setting the *EJO* and *PROGRESS*, as leading—although the latter in a more restricted manner due to the limited number of published RCTs over the years—might reflect persistent efforts made by the editorial team and publishers over the years in those journals. Besides these two journals, *the JOOF and the OCR* have also adopted the requirement to publish a data availability statement, and five further journals in the sample are currently recommending it (Supplementary Table 2).

The low rate of data and code sharing in orthodontic RCTs risks introducing bias and hinders critical assessment by clinicians and students. While high-impact journals like *The Lancet* and the *NEJM* demonstrate better transparency standards with more than two-thirds including data sharing statements, only approximately 6% provided their data openly upon publication [29]. This shows that such impactful journals in the medical field also bear witness to similar transparency deficiencies, however, to a lesser extent. The gap may lead to misguided decisions based on incomplete or inflated evidence, emphasizing the need for greater access to anonymized raw data to ensure informed, evidence-based decisions.

The results show that there has been a notable increase in data sharing practices among orthodontic RCTs over the past 3 years, although room for considerable improvement is profound. The rise may indicate growing support from editors and journals for better research practices, or even more knowledgeable authors and researchers. A recent study found that about 60% of dental journals now recommend data sharing, however, these guidelines are not mandatory even for clinical trial submissions [30].

The open publication or registration of RCT protocols in peer-reviewed journals or registries has been identified to be an important transparency indicator for credible research in both biomedicine [19] and orthodontics [19, 31], protecting against inconsistencies in reporting and misconduct [11, 20]. Incorrect reporting can significantly affect clinical decision-making, as treatment choices rely on the available evidence. Therefore, without research transparency, the risk of research waste in orthodontics may increase [15]. In the present study, although a greater number of registered RCTs followed data sharing practices, this effect was not evident through the multivariable model. A larger sample, or a more even distribution of other publication parameters, might have revealed a clearer association between registration and data sharing perspectives.

In line with the above, biomedical empirical research has revealed the link between the lack of registration of studies and reporting bias or publication biases favouring positive results in the final reports [32]. The absence of data sharing statements or open registration of RCT data and statistical code impedes reproducibility and replicability, allowing issues like data manipulation and selective reporting of significant results to persist. This bias appears less prevalent in orthodontics, where the reporting of positive versus negative non-significant findings has lately been more balanced in RCTs [20], while the present study does not confirm any association between significant findings and data sharing. Our findings on code or script sharing are particularly concerning, however, they align with broader trends in biomedicine [10]. Following findings from a recent study in oral health, none of the RCTs included from 15 orthodontic journals in the present report had their statistical code openly provided, at least regarding the primary research outcome [27].

Previous research has indicated that industry-sponsored projects more often report favourable efficacy results [23]. Our study confirmed a link between data sharing practices and funding. While the majority of the RCTs in this sample were not funded or did not reveal any funding information, for those externally funded, the likelihood of reporting on data sharing was higher. Externally funded RCTs are more likely to report data sharing in journal publications due to specific requirements set by funding agencies, such as the National Institutes of Health or the European Union, which promote transparency and open science. These trials face greater accountability and scrutiny, with funders often mandating data sharing to maximize research impact and ensure ethical practices [11, 13].

For outcome-related predictors, the outcome type (efficacy vs. safety) also revealed differences in data sharing practices. Studies focussing on efficacy outcomes were more likely to include data sharing statements compared to those focussing on safety. Efficacy outcomes are generally more prevalent in biomedical research as authors seek to confirm the dynamics and strength of a newly developed intervention or product and as such, investigators of these types of outcomes might be more familiar with robust and transparent research practices. However, one might consider safety outcomes as equally or even more important for the identification of adverse effects and patient-centred outcomes [28].

There is a strong need for stricter adherence to transparent research practices among authors, reviewers, and editors, involved in orthodontic research, coupled with changing mentality and education. Trial pre-registration, immediate dissemination of study findings even as pre-prints, and changing journals’ policies from optional to mandatory inclusion of clear data sharing statements with further monitoring are some of the proposed plans.

This study represents the most large-scale, systematic evaluation of data sharing practices across the entirety of indexed orthodontic journals, focussing on transparency indicators in RCTs. It serves as a foundation for journals and editors to shift their perspectives and policies and emphasize transparency in their disseminated evidence. However, the limitations of this work must be acknowledged. First, the study relies on the self-reported data sharing intentions of the authors, which may not fully reflect actual practice. Our methodology was based solely on the information provided in the final published RCT reports, which is, however, usually the case in meta-research. This might lead to an overestimation of actual data sharing practices, as intentions may not always translate into action. In addition, the exclusion of non-English language studies may limit the generalisability of our results. Moreover, the variability in data sharing policies across journals further complicates the analysis, as different journals might have varying requirements and incentives for data sharing. It was out of the scope of the present work to capture how these differences impact authors’ likelihood of including data sharing statements [30]. On the same grounds, we did not account for potential recent modifications in journal or funder policies that may not yet have been reflected in the analysed studies, particularly those published earlier in the study period; we recorded the current state of the art regarding data sharing reflecting authors/investigators perspectives, rather than journals’ policies. There is also the potential for unmeasured predictors—such as authors’ prior experiences with data sharing and the complexity or sensitivity of the data—that might impact the results.

Future research should focus on identifying barriers that prevent orthodontic researchers from sharing their data and assessing whether mandatory data sharing policies lead to improvements in research quality and transparency. Insights into why authors choose not to share their data and understanding the reasons behind non-sharing—such as concerns about data misuse, intellectual property issues, or lack of resources—could be crucial for addressing barriers to transparency.

## Conclusions

Less than 20% of published orthodontic trials include a positive data sharing statement and only about 2% openly provide their data with publication. Key predictors influencing data sharing practices were the year of publication, efficacy outcomes, and external funding. The results highlight the need for continued emphasis on these aspects to improve data sharing and thus transparency in clinical orthodontic research.

## Supplementary Material

cjae064_suppl_Supplementary_Tables_1-2

## Data Availability

The data underlying this article are available in the Open Science Framework, at https://osf.io/avt5b/.
