# Supplementary material for: Indicators of transparency and data sharing in scientific writing in published randomized controlled trials in orthodontic journals between 2019 and 2023: an empirical study
Source: Eur J Orthod. 2024 Nov 21;46(6):cjae064. doi: 10.1093/ejo/cjae064 (PMC11579657; doi:10.1093/ejo/cjae064)
Supplement: cjae064_suppl_Supplementary_Tables_1-2 [file cjae064_suppl_supplementary_tables_1-2.docx]

**Supplementary Material**

**Supplementary Table 1.** List of Registries reported in the subsample RCTs with registered protocols (n=195).

| **Registry** | **N** | **Percentage (%)** |
| --- | --- | --- |
| Australian New Zealand Clinical Trial Registry | 7 | 3.6 |
| Brazilian Registry for Clinical Trials- ReBEC | 7 | 3.6 |
| Chinese Clinical Trial Registry- ChiCTR | 4 | 2.1 |
| Clinical Research Information Service (CRIS) Korea Republic | 1 | 0.5 |
| ClinicalTrials.gov | 108 | 55.4 |
| FoU i Sverige | 3 | 1.5 |
| German Clinical Trials Register- DRKS | 7 | 3.6 |
| Indian Clinical Trials Registry- CTRI | 20 | 10.3 |
| Iranian Registry of Clinical Trials | 18 | 9.2 |
| ISRCTN | 13 | 6.7 |
| Northern Ireland Hub for Trials Methodology Research- SWAT Repository | 1 | 0.5 |
| Pan African Clinical Trials Registry | 2 | 1.0 |
| Thai Clinical Trials Registry | 4 | 2.1 |
| **Total** | **195** | **100.0** |

**Supplementary Table 2.** Current requirements for data availability statements across all indexed orthodontic journals (n=16).

| **Journal** | **Data Availability Statement paragraph in author guidelines** | **Data Availability Statement** |
| --- | --- | --- |
| American Journal of Orthodontics and Dentofacial Orthopedics (AJODO) | To foster transparency, we **encourage** you to state the availability of your data in your submission. This may be a requirement of your funding body or institution. If your data is unavailable to access or unsuitable to post, you will have the opportunity to indicate why during the submission process, for example by stating that the research data is confidential. The statement will appear with your published article on ScienceDirect. For more information, visit the Data Statement page. | encouraged |
| Australasian Orthodontic Journal | no information provided | no information |
| Clinical and Investigative Orthodontics (formerly known as Orthodontic Waves) | Data availability statement. **If there is a data set associated with the paper, please provide information about where the data supporting the results or analyses presented in the paper can be found.** Where applicable, this should include the hyperlink, DOI or other persistent identifier associated with the data set(s). Templates are also available to support authors. Data deposition. If you choose to share or make the data underlying the study open, please deposit your data in a recognized data repository prior to or at the time of submission. You will be asked to provide the DOI, pre-reserved DOI, or other persistent identifier for the data set. | encouraged |
| Dental Press Journal of Orthodontics | no information provided | no information |
| European Journal of Orthodontics | Data availability statement The inclusion of a **data availability statement is a requirement** for papers published in the Journal. Data availability statements provide a standardized format for readers to understand the availability of original and third-party data underlying the research results described in the paper. The statement should describe and provide means of access, where possible, by linking to the data or providing the required unique identifier. | **required** |
| International Orthodontics | To foster transparency, we **encourage** you to state the availability of your data in your submission. This may be a requirement of your funding body or institution. If your data is unavailable to access or unsuitable to post, you will have the opportunity to indicate why during the submission process, for example by stating that the research data is confidential. The statement will appear with your published article on ScienceDirect. For more information, visit the Data Statement page. | encouraged |
| Journal of Orofacial Orthopedics | This journal follows Springer Nature research data policy. Sharing of all relevant research data is strongly encouraged and authors **must** add a Data Availability Statement to original research articles. [...] Data availability statements: All original research **must include a data availability statement**. This statement should explain how to access data supporting the results and analysis in the article, including links/citations to publicly archived datasets analysed or generated during the study. | **required** |
| Journal of Orthodontics | The Journal is committed to facilitating openness, transparency and reproducibility of research, and has the following research data sharing policy.Subject to appropriate ethical and legal considerations, authors are **encouraged** to: Share your research data in a relevant public data repository Include a data availability statement linking to your data. If it is not possible to share your data, use the statement to confirm why it cannot be shared. | encouraged |
| Journal of the World Federation of Orthodontists | no information provided | no information |
| Orthodontics and Craniofacial Research | Orthodontics & Craniofacial Research **expects** data sharing. All accepted manuscripts will **need to publish a data availability statement** to confirm the presence or absence of shared data. The journal expects authors to share the data and other artefacts supporting the results in the paper by archiving it in an appropriate public repository. Authors should include a data accessibility statement, including a link to the repository they have used, in order that this statement can be published alongside their paper. Review Wiley’s Data Sharing policy where you will be able to see and select the data availability statement that is right for your submission. If you have shared data, this statement will describe how the data can be accessed, and include a persistent identifier (e.g., a DOI for the data, or an accession number) from the repository where you shared the data. Sample statements are available here. If published, statements will be placed in the heading of your manuscript. | **required** |
| Progress in Orthodontics | All manuscripts **must** include an ‘Availability of data and materials’ statement. Data availability statements should include information on where data supporting the results reported in the article can be found including, where applicable, hyperlinks to publicly archived datasets analysed or generated during the study. By data we mean the minimal dataset that would be necessary to interpret, replicate and build upon the findings reported in the article. We recognise it is not always possible to share research data publicly, for instance when individual privacy could be compromised, and in such instances data availability should still be stated in the manuscript along with any conditions for access. | **required** |
| The Angle Orthodontist | no information provided | no information |
| APOS Trends in Orthodontics | Data Sharing Policy NIH funded researchers, please follow the NIH guidelines on Data sharing as given at: https://grants.nih.gov/grants/guide/notice-files/NOT-OD-21-013.html | only for NIH |
| Korean Journal of Orthodontics | no information provided | no information |
| Seminars in Orthodontics | Linking to the data underlying your work increases your exposure and may lead to new collaborations. It also provides readers with a better understanding of the described research. If your research data has been made available in a data repository there are a number of ways your article can be linked directly to the dataset: Provide a link to your dataset when prompted during the online submission process. For some data repositories, a repository banner will automatically appear next to your published article on ScienceDirect. You can also link relevant data or entities within the text of your article through the use of identifiers. Use the following format: Database: 12345 (e.g. TAIR: AT1G01020; CCDC: 734053; PDB: 1XFN). | encouraged |
| Turkish Journal of Orthodontics | no information provided | no information |
